# On Living Worms in Living Men; a Treatise for the Use of Practical Physicians, &c.

**Published:** 1821-01

**Authors:** 


### CRITICAL ANALYSIS

OF

### RECENT PUBLICATIONS, IN THE DIFFERENT BRANCHES OF MEDICINE AND SURGERY,

### In the Literature of Foreign Nations.

---

Πατρίδες ἄρα
Ανδράσιν, ὖ πατραῖς ἄνδρες, ἀγαλλόμεθα.

---

*Ueber lebende Würmer im lebenden Menschen; ein Buch für ausübende Aertze.* Von Dr. Bremser. 4to. seit. xii. und 284. Wien, 1819. —i. e. *On living Worms in living Men; a Treatise for the Use of Practical Physicians, &c.*

[In continuation from vol. xliv. p. 427.]

THE sixth chapter of this work treats of the medicines which have been employed for verminous diseases. Amongst the immense number of those which have, at various times, been proposed as remedies of a specific character, there have been but few which have preserved, amongst the generality of practitioners, the reputation they had once acquired. The versatility of opinion on this subject is attributed by the author, 1°, to the reputed remedies having been employed without, at the same time, any regard being paid to the cause of the generation, or the presence, of the worms, and without efforts to remove it; 2°, to their having been often used where the presence of worms was only suspected from some general symptoms; or where, on the expulsion of worms, the morbid phenomena have been attributed to the presence of those animals, when they really proceeded from some other cause; 3°, to the accidental evacuation of worms, during the treatment of some other diseases, having been considered as the effect of the medicines employed. Some attempts have been made to settle these doubts, as far as the immediate effect of several medicines on the life of the worms themselves are concerned, by exposing the animals, when out of the body, to the influence of the supposed remedies: but it is obvious that nothing very precise, or applicable to practice, can be drawn from experiments made under circumstances so different from those which concern the medical practitioner.

The author divides the remedies which may be administered to human beings for the treatment of verminous diseases, into the following classes.

1°. *Mechanical anthelminthics.*—To this class may be referred the filings or powder of tin; cowhage; pulverized charcoal; and raw carrots, proposed by Rosenstein. Dr. Bremser does not doubt that these substances have produced the expulsion of worms; but he does not favour the use of them, unless combined with other remedies, because, though they may facilitate the removal of the animals, they have no power to remedy the disorder on which the existence of the worms depends; the removal of which is the principal indication in the cure. Notwithstanding these objections, the filings of tin, and especially cowhage, will be found very useful in practice, for their efficacy in causing the evacuation of worms. On speaking on this subject to two physicians who have practised at the same Dispensary (where diseases of children are witnessed to a great extent,) for several years, one of them said that cowhage alone will generally prove a remedy for worms in children, without any other medicine, either combined with it or subsequently administered: the other said he always gave at the same time calomel, or some other medicine, with the view of restoring the healthy state of the intestines. The cowhage is most conveniently administered in the form of an electuary.

2°. *Specific anthelminthics:* of which he enumerates,—*a, cold water* in large quantities and almost constantly repeated doses, proposed by Rosentein and Pallas, to be drank after having taken a purgative. Dr. Bremser supposes that the cold water causes their expulsion by the shivering which it produces, on being drank in large quantities, the disagreeable impression of which on the worms causes them to leave the intestines: he also thinks that the animals absorb some portion of it, by which they become tumid, torpid, and incapable of resisting the afflux of the quantity of water drank by the patient. Salt water, he says, acts still more efficaciously.

*b. Valerian root.*—This forms the principal ingredient of the well-known vermifuge of Stork; and is, he considers, a useful remedy in many cases, especially where the nervous system is much affected.

*c. Onions* and *garlic,* are reputed remedies, but are not at all certain in their efficacy. Several instances are, however, recorded, in which they seem to have been efficacious, for tænia especially. Brera recommends a liniment made of an ounce of bruised garlic and a drachm of camphor dissolved in six ounces of sulphuric ether, to be applied to the abdomen; especially in conjunction with some others that will be hereafter described.

*d. Semen santonici,* and the seeds, or rather the mature flowers, of the *tanacetum vulgare.*—The author remarks, that the fine powder of those substances soon loses great part of its virtue: he thinks the seeds only grossly powdered are more efficacious, in which form they seem also to act beneficially in a mechanical manner. Brera believes these medicines to possess considerable efficacy.

*e. Conferva helminthochortos,* has been sometimes used with apparent advantage in quantities of from a scruple to half a drachm, or rather of an infusion to the extent of two drachms in four ounces of water, daily.

*f. Chenopodium anthelminthicum,* (Jerusalem oak.)—The seeds of this are frequently used in America. Chalmers says, that it is the active ingredient of the anthelminthic which has been so famous in North America.

*g. Angelica bark.*—An ounce of this is boiled in three pints of water down to one pint; one or two ounces of which are to be taken every morning. This medicine is apt to produce griping; but it produces the expulsion of worms in large quantities when they exist in great numbers.

*h. Grana tiliæ* (linden-seeds), are rather purgatives than proper anthelminthics.

*i. Spigelia anthelmia* and *marilandica.*—The former of these has been long employed in America: the latter was praised by BERGIUS as the best of vermifuges; and Dr. LINING, of South Carolina, favours its use. Both produce narcotic effects, and at the same time copious evacuations from the bowels. They are violent medicines, and may be spared without much loss, according to Dr. Bremser. The spigelia anthelmia has, however, been strongly recommended by BROWN, ROSENSTEIN, and some other physicians; and BRERA says, that he has frequently prescribed it with the greatest success.

*k. Surinam root*, or the *geoffrea surinamensis*, (worm-bark tree.) —SCHWARTZE says, a very efficacious tincture may be prepared by digesting two ounces of the root with four ounces of rectified spirit of wine and two pints of water for six days; the mixture is then reduced, by evaporation by a gentle heat, to one pint. For two days, the patient is to take two table-spoonfuls in the morning fasting, and repeat the dose twice after intervals of an hour; on the third day, he is to take the rest, by tea-cupfuls; on the fourth, a purge of jalap and calomel. The author does not think that much reliance should be placed on this medicine. Brera, on the contrary, says that he has employed it, and known it employed, many times, with efficacy against ascarides, both vermiculares and lumbricoides. He says, it is more beneficial when combined with valerian.

*l. Cevadilla*, veratrum sabadilla.—The seeds were first employed, internally, by LOESER, and afterwards by SCHMUCKER, in cases where NOUFFER's remedy had failed. They should be cautiously employed, as they possess very acrid qualities. Children but rarely bear more than three or four grains, daily. Schmucker obtained great success in his treatment; but it should be remarked, that he used at the same time calomel and jalap. He thought clysters of a decoction of the seeds with milk, beneficial, in conjunction with other means, in cases of ascarides vermiculares.

*m. The green rind of walnuts*, in the form of infusion or extract, has been much esteemed in modern as well as in ancient times. It may be given in doses of one or two drachms, in infusion or decoction; or two drachms of its extract may be dissolved in half an ounce of cinnamon-water, and from fifteen to thirty drops of the mixture given to children twice a-day.

*n. Assafœtida*, is sometimes used, in the form of a pill.

*o. Camphor*, was particularly recommended by PRINGLE and MOSCATI for the *ascarides lumbricoides.* It may be given in doses of from a grain to a scruple, in pills, with or without assafœtida, or infusion of valerian. It would appear, from the observations of PALLETA, that this substance is particularly noxious to the worms; and that, when administered by way of clyster, in children who could not be made to take it by the mouth, it has occasioned the worms to retire from the lower to the upper part of the intestines; where they have, nevertheless, been destroyed, when the use of the remedy has been vigorously pursued in the same manner. It is thought, too, that this medicine will prevent the development of any ova which may be left in the intestines. Brera says, that he has always used it with the greatest success.*

*p. Polypodium filix mas*, has continued to possess, down to the present day, the reputation it had in the time of GALEN. It is said by some authors to be almost a certain remedy against the tænia lata, (*bothriocephalus;*) but not, according to Dr. Bremser especially, so certain against the tænia cucurbitina, the prompt regeneration of which it does not prevent. It is the principal ingredient in the famous remedy of NOUFFER, said to be an infallible specific against the tænia. It may be given in doses of from one to three drachms to an adult, in powder or infusion.

*q. Petroleum.*—This has been said to be commonly used with success in Egypt; given in doses of twenty or thirty drops, for three mornings in succession; after which time a purgative is administered. LARREY, however, says that tænia is but rarely seen, and that petroleum is unknown, in Egypt. It has been, however, a famous remedy at Montpellier.

*r. Oil of turpentine.*

*s. Cajeput oil;* recommended by RUDOLPHI.

*t. Dippel's animal oil*, taken in the dose of from three to ten drops, three times a-day, in broth, is an efficacious anthelminthic, according to Rudolphi.

*u. Chabert's empyreumatic oil*, is, the physiologist just named says, preferable to the foregoing. It is prepared in the following way:—Take fetid oil of hartshorn, one part; oil of turpentine, three parts; mix them well together, let them stand for four days; then distil the mixture in a sand-bath, and draw off three-fourths of the liquor. It is to be kept in a phial with a glass stopper, tied over with a piece of bladder.

*v. Fluid mercury.*—Water boiled over mercury is an old domestic vermifuge. Experience has, however, proved that the water takes up only the impurities, or a little of the oxide which exists on the surface of the quicksilver of commerce. Fluid mercury has no specific action on worms.

*w. Muriate of barytes*, has been much recommended, (since its internal use was advocated by CRAWFORD and CLARK,) in verminous diseases, by several physicians, as one of the most powerful remedies against ascarides vermiculares. Great caution is required in its administration.

The many remedies which have been proposed for external application, are considered by the author to have but little utility; though, he thinks, some of them may act beneficially by their influence on the nervous system, and especially on the abdominal plexuses. In this opinion many other experienced practitioners are at variance with

---

* See the xxvth volume (p. 25) of this Journal, for a curious instance of the efficacy of this remedy.

him. We know a physician to a public institution, where his practice for some years has been very extensive in the diseases of children, who says that the external application of oil of turpentine to the abdomen, is but little less efficacious than its internal exhibition in a great proportion of cases; and Frank, Weikard, and Alibert, have regarded the same mode of using the anthelminthics in general in as favourable a manner.*

The third class of anthelminthics consists of *Purgatives*.

The chief use of medicines of this class, is to expel the worms destroyed or collected together by the foregoing remedies. Several authors have recommended, especially, the sulphate of soda and of potash, muriate of soda; tartarized antimony; calomel; muriate of barytes; the fat oils, especially nut and castor oil. Dr. Bremser prefers senna and jalap to any of them. Calomel, he thinks, is a vermifuge only in consequence of its being a purgative.

4°. *Tonics*.—These may be employed to restore the strength of the intestinal tube, that may have been destroyed by the remedies employed for the removal of the worms. The author recommends particularly bitters and iron. Professor Brera is disposed to consider tonics as of considerable utility for the immediate relief of the verminous disorder itself, which he regards as a state of asthenia of the digestive organs. He advises the administration of them with purgatives in many instances.

Dr. Bremser says nothing respecting the use of emetics, which have been very strongly recommended by some physicians. Prof. Brera strongly reprobates the practice, as he does the exhibition of violent purgatives, which, although they may occasionally produce the expulsion of worms, often tend to favour their re-production, by increasing the morbid state which is the cause of their presence in the intestines.

The agency of culinary salt as a remedy against worms, is not noticed by Dr. Bremser. On this subject we refer to a paper by Mr. Marshall, published in this Journal for May 1818; and to Heberden's paper in the first volume of the Transactions of the College of Physicians. We shall notice the oil of turpentine hereafter, in a particular manner.

So far our observations have related principally to the remedies for verminous disorders in general; but remedies which are efficacious for one species, ascarides for example, are often of no utility for others, as for the tæniæ. This leads us to the seventh chapter, in which the

---

* Brera recommends the following liniments, for friction on the abdomen, as of great utility, especially in children.

1°. A drachm of ox-gall, and the same quantity of Castile-soap, to be made into a liniment with a sufficient quantity of oil of tansy.

2°. Two ounces of ox-gall, half an ounce of socotrine aloes, and the same quantity of powder of colocynth, are to be digested for twenty-four hours, in a warm place, in a sufficient quantity of gastric juice or saliva, and the mixture to be made into a liniment with lard.

Alibert says, that the gastric juice (if such a fluid exist) is devoid of utility; for that he has obtained equally advantageous results from the application of the remedies without it.

author discusses the particular treatment of the several species of worms.

1°. The *tricocephalus dispar.*—We stated, in a former part of this Review, that there was no evidence of the existence of this worm in the living body, for that no practitioner had mentioned having seen them expelled during life. Dr. Bremser, however, we now find, says that it has once occurred to him to remark a worm of this species, which was evacuated from the anus with a multitude of ascarides lumbricoides and vermiculares. Until more instances of this kind have been witnessed, this species of worm cannot be regarded as an object of medical treatment.

2°. The *oxyures vermiculares,* are commonly productive of distress by the irritation which they excite, though sometimes thousands exist together without producing the slightest inconvenience. The most common effect from them is an insupportable itching about the rectum, which is joined in children with much nervous disturbance. The destruction of these worms is not, in general, very easily effected: not only in consequence of the great rapidity with which they are re-produced, but also from the mucous secretion of the intestine serving to defend them against the means employed for their destruction. Dr. Bremser has found very useful, though often only as a palliative, the following mode of treatment:—In the first instance, in order to remove the worms from the upper part of the large intestines, he directs to be taken, morning and evening, a coffee-spoonful of the electary marked No. 1, in the collection of formula which will be presently adduced; with which he joins a sufficient quantity of jalap to purge the bowels gently. Besides this, he orders two clysters daily, prepared according to formula No. 2, taking care that the lower intestines have been evacuated previously to their administration. In patients of a very irritable habit of body, a spoonful of fresh ox-gall should be added to each clyster. The patient should endeavour to retain the clysters as long as possible. This practice is to be continued for several weeks. A clyster of olive-oil will procure immediate relief from the pruritus occasioned by these worms.

Professor Brera says, that we may sometimes relieve the irritation produced by these worms, when they are situate near to the anus, by passing into the rectum a piece of bacon tied to a string: this is to be withdrawn after a little while, when a considerable number of ascarides will be found attached to it. The practice may be repeated until all the worms are brought away.

Clysters of decoction of Surinam-root, assafœtida, the veratrum sabadilla, aloes and lime-water, salt-water, and semen santonici (especially recommended by Mr. Charles Clark), are also occasionally efficacious in bringing away those collected about the anus: but, when there is much irritation of the intestine, castor-oil and mucilaginous liquids are preferable for this purpose. Tobacco was once strongly recommended with the same intention; but Heberden stated that it was injurious rather than beneficial, and it does not seem to have been much employed.

The more efficacious of the remedies administered by the mouth, generally employed, are the spirit of turpentine, camphor, muriate of barytes, cowhage; and, in certain cases, especially when the functions of the digestive organs are much deranged, calomel; with, or without, various tonics, as the muriatic tincture of iron, and bitters, or assafœtida. The latter medicines are generally especially useful after the worms have been evacuated, to prevent their re-production.

3°. The *ascarides lumbricoides.*—In the choice of the numerous remedies proposed for these worms, it is of essential importance that those should be preferred which tend not only to destroy them, but to remove the causes of their generation, or their presence, in the intestines. Dr. Bremser, when he suspects them to exist, puts in practice the following measures. He orders a child to take, morning and evening, a dessert-spoonful of the electuary No. 1: after three or four days, the intestinal evacuations begin to be more frequent and more copious than ordinary, and commonly much mucus, and perhaps a few worms, are expelled. Where the latter effects do not take place, he directs the dose of the medicine to be increased, or to be taken more frequently. If, with this addition, the desired amendment is obtained, he advises the electuary to be continued, moderating the dose, so as to produce only some augmentation of the evacuation of feces and intestinal mucus; administering also, from time to time, the mild purgative No. 3. According to the author, it is a matter of indifference whether or not worms are evacuated during the use of these means: sometimes they are not passed until all the signs of the verminous disorder have disappeared. In patients of a leucophlegmatic habit of body, in order to prevent a prompt relapse, he orders for some time a few drops of the mixture No. 4.

In respect to diet, the patient should abstain from the extensive use of farinaceous substances, legumens, and of fat meat; and should eat, in preference, dry bread. Dr. Bremser has found this treatment so efficacious, that he has not had occasion to employ any other means.

The measures which have most generally been found efficacious against these worms, do not differ materially from those proper in the treatment of ascarides vermiculares; only, as the lumbricoides inhabit higher parts of the intestines, medicines administered by the mouth are more likely to be successful, and clysters less so, than with the latter species. We have nothing of importance to add to what we said when treating of the vermiculares, and in the express review of the particular remedies. We may, however, mention here that the cowhage is in no cases so generally efficacious as in the treatment for lumbricoides.

4°. The *tænia lata* and *cucurbitina.*—The symptoms in the case of the presence of either of these worms, do not differ remarkably from those which arise from the ascaris lumbricoides: the expulsion of some of the joints of them is the only means of ascertaining, with certainty, their existence. In general, the ordinary vermifuges fail to produce their evacuation; and hence it is that such a multitude of medicines have been tried. It is still more difficult to prevent their regeneration. Dr. Bremser says, that he has never known the latter species of tænia expelled by the ordinary remedies. He has had, he says, in the course of ten years, more than five hundred patients affected with tænia cucurbitina, amongst whom two were children of about a year and a half old. Only four of all those patients were obliged to have recourse a second time to the use of the remedy which he employed; and in one only had the worm been re-produced at the end of two years. The following is his mode of treatment, which he considers to be as efficacious in the cure of the tænia lata as in that of the cucurbitina. He commences with the electary No. 1, which is to be taken as directed when speaking of the ascaris lumbricoides. On the quantity prescribed being consumed, the patient is to take, having his mouth full of water at the instant, two coffee-spoonfuls of the oil No. 4. This medicine is much more ungrateful to the smell than to the taste. It is better that the patient should not rinse his mouth afterwards, to remove the particles of the oil adhering about it: he should only have his mouth previously full of that liquid in order to facilitate the passage of the oil to the stomach. He may, however, chew a piece of cinnamon or some cloves. If this dose should produce irritation or head-ach, it should from the first be diminished; and, when it excites nausea if taken fasting, it may be administered an hour or two after breakfast. The ardor urinæ which sometimes supervenes on the use of this remedy, may be removed by some oily or amygdaline emulsion. By proceeding in this way, the patient, at the end of ten or twelve days, will have taken from two to three ounces of the medicine. He should then take the purgative No. 3; and after this resume the use of the oil. Generally four or five ounces should be consumed; and, where the worm has resisted the influence of other medicines, six or seven ounces may be requisite. It will be prudent, however, to continue the treatment for some time longer, partly to remove the disposition to the generation of worms, and partly to prevent the development of the ova in the intestines which may have been left by the worm which has been expelled. All secondary treatment is superfluous, excepting in the cases where there is a predominant disposition to the formation of mucus in the intestines and the generation of worms; against which the drops No. 5, may be advantageously employed. The diet during the treatment should be subjected to similar restrictions to those mentioned as proper in cases of ascarides. The worm, under this treatment, generally comes away in pieces, sometimes almost like strings of mucus; but the proper form of the animal can commonly be recognized by careful examination. When no more portions have been evacuated by the end of three months, the author says we may be certain that the whole has been destroyed. When new fragments appear at the end of two or three years, as is sometimes seen, it must be considered that a new worm has been generated.*

---

* The following are the formulæ alluded to in the foregoing observations:

No. 1.—℞. Sem. cinæ s. tanaceti, contus. ℥j.

Pulv. rad. valerianæ, ʒij.

—— jalapii, ʒss. ad ℈ij.

Potassæ sulphatis, ʒj ss. ad ʒij.

Oxym. scillæ, q. s. ut fiat electuarium.

Being satisfied with this mode of treatment, and not placing any confident reliance on the other means which have been proposed as remedies for tæniæ, Dr. Bremser passes them over almost unnoticed: we shall, therefore, deviate a little more than ordinary from the running-base with which we have accompanied the author, and give an *arbitrio* to fill up the vacancy which he has left in the piece.

We should first remark that several cases have occurred in which very long tæniæ have been evacuated whole, after the use of cold water in the way recommended by Rosenstein; and still more when various mineral waters have been thus employed. It is, probably, from their having been drank in the way just designated, that the waters of *Chad's Well,* at Battlebridge, were a few years since so celebrated a remedy for tænia.

The most powerful, and most constantly efficacious, of all the remedies ordinarily employed for tænia, is the oil of turpentine; the introduction of which into practice is due to Dr. Fenwick. The utility of this medicine is, however, too well known to English practitioners to render it necessary to dwell upon it here.

The remedy of Nouffer, the formula for which was purchased for a large sum of money by the king of France, and which, towards the middle of the last century, was so much celebrated in France, consisted principally of the *polypodium filix mas:* but, according to the report of the French physicians who were appointed to investigate its effects, it is of no utility against the tænia cucurbitina, though generally a remedy for the tænia lata. Dr. Harrenschwaud had previously employed the same remedy in Germany, and had acquired great reputation for the success of his practice. Brera, however, relates some cases in which the administration of this medicine was followed by the

---

No. 2.—℞. Herbæ absynthii

Rad. valerianæ, ā ℥j.

Sem. tanaceti

Cort. aurant. ā ℥ ss.

To be reduced to a coarse powder, and mingled together. Two table-spoonfuls are to be put in two pints of boiling water, and the mixture suffered to stand in a close vessel for twelve hours; it is then to be strained through cloth, and will serve for two clysters; to each of which a spoonful of fetid oil of hartshorn is to be added.

No. 3.—℞. Pulv. jalapii, ℈j.
Fol. sennæ, ʒss.
Potassæ sulphatis, ʒj. M. f. Pulvis, in tres quatuorve partibus dividendus. The patient is to take the half of one of these powders every half-hour, or every hour, until the purgative effect is produced.

No. 4. is the vermifuge oil of Chabert, the mode of preparing which was stated when we passed in review the different remedies for worms. Dr. Bremser is the first person who proved that this medicine might be safely and effectually administered to human beings: it had previously been given only to animals.

No. 5.—℞. Tinct. alöes comp.* ℥j.
Tinct. martis. pom. ℥j.
Elixir vitrioli mynsichti, ℥ ss. M. To be taken in doses of ten, twenty, thirty, or more drops, three times a-day, in a glass of water or wine.

* This is according to the *Pharm. Austriaco-provincialis,* and is the same with the *elixir proprietatis* of Paracelsus, which does not differ materially from the *T. aloes comp.* of the London and Dublin Pharmacopœias.

evacuation of the armed tænia; but it seems that this occurred when the worms were young, and not able to attach themselves firmly to the intestines by the crotchets in their head.

The oleum ricini, taken in doses of two or three ounces, obtained much estimation in the practice of ODIER of Geneva, as a remedy for the tænia lata only. It appears occasionally to kill the worm, and at the same time produce its expulsion from the bowels. Brera says, that it is sometimes efficacious against the armed tænia.

Tin, in filings especially, as already noticed, has acquired much credit as a remedy for both the species of tænia. The reputation of this medicine seems to have declined of late years, particularly in England. We have, ourselves, known very severe colic produced by it; but we should, at the same time, state that we have frequently known it produce the evacuation of tænia of both species.

An apothecary of Berlin, named MATHIEU, acquired much reputation for many years for the treatment of tænia, and received great honours from William III. in consequence of his successful practice. His remedy was a combination of tin-filings, male fern, santony seed, jalap. scammony, gamboge, and sulphate of potass.*

Another remedy, which has been proposed with much confidence, is the pomegranate root, given in the form of decoction; and the evidence in its favour is such as to place it amongst those most deserving of trial. (See vol. xxxii. of this Journal.)

Some caution is necessary in the management of the case when the worm is partially expelled. It might appear that the animal might be wholly withdrawn by pulling at it; but, generally, either a great deal of pain in the intestines is produced by the attempt, or the worm is broken. Instead of attempting this, Brera advises that a thread should be tied around some part of the expelled portion: the worm retires a little into the intestines when this is effected, but soon afterwards passes wholly from them.

In the eighth chapter, Dr. Bremser treats of worms which inhabit other parts of the body of man than the intestines. These are—

1°. The *filaria dracunculus*, *filaria medinensis* (GMELIN). We must pass over what the author says about the history, and the description, of this worm, as it relates to natural history rather than to pathology; and he does not advance any thing novel of importance, either in regard to the symptoms which ensue from it, or to its treatment. Dr. Bremser is, however, almost singular in the opinion that it is, like intestinal worms, primitively generated in the human body. For particular information on this subject, we refer the reader to the paper of Dr. CHISHOLM, published in the 42d Number of the Edinburgh Medical and Surgical Journal, or to the abstract inserted in the 197th of our own.

2°. *Hamularia subcompressa.*—Dr. Bremser is not perfectly convinced of the presence of this genus in the human body, which TREUTLER had attempted to establish as existing in the bronchial glands, where he

* See the fifth volume, page 398, of this Journal, for a particular account of the mode of using this remedy.

stated he had found a considerable number in the year 1790. But, though so long a period has since elapsed, no other persons seem to have observed them.

3°. *Strongylus gigas.*—This worm has been found but rarely in man: it inhabits the kidneys, and perhaps also the adjacent muscles. It has often been stated by medical practitioners that worms have been passed with the urine; but, in by far the greater proportion of cases, the supposed worms have been lymphatic concretions, or else they have passed from the intestines into the bladder.

Amongst the worms which appear to be nourished by means of general absorption by their surface, there occur in man:

1°. The *distoma hepaticum,* which inhabits the gall-bladder, and perhaps also the liver. Dr. Bremser has never had an opportunity of seeing it in man. This is the common *hydatid*, which is seated in the liver of inferior animals, especially in sheep and pigs.

2°. *Polystoma pinguicola*, discovered by Treutler in fat about the ovary of a woman.

According to the author, the term *hydatid* appertains properly to those vesicles, full of a limpid fluid or more dense matter, which are found in the bodies of man and animals, and which, without any connection with the surrounding parts, are enclosed in a particular capsule appertaining to the organ in which the vesicle is seated. This definition, he says, will enable us to distinguish true hydatids from a varicose dilatation of the lymphatics, or distention of the cellular tissue. Hitherto only the two following species have been discovered in man.

1°. The *cysticercus cellulosæ.*—It inhabits the cellular texture of the muscles and the brain. Its existence in domesticated pigs has been long observed; but Werner was the first who remarked it in man, and it is asserted to have been present in almost the whole of the muscles of the body. Dr. Bremser has nothing of importance to say respecting the etiology, pathology, diagnosis, or therapeutics, of these worms. Nothing, indeed, seems to be known on the subject.

2°. *Echinococcus.*—Rudolphi, as we have already stated, makes two species of hydatids,—those having, and those not possessing, peculiar life; whilst Dr. Bremser maintains that it is only those which are enclosed in a particular capsule, not attached to this capsule nor to the organ in which they are seated, that are real worms; and to these he appropriates the term hydatid. None of those animals, of this genus, having a sort of crown with crotchets and a sucking mouth, has been found in man; but distinct vesicular worms, of a spherical figure, have been observed in all parts of the human body excepting the intestines. Dr. Bremser's own observations have led him to agree with several former authors, that hydatids, of the genus echinococcus, have been found in the uterus and placenta, both with or without a fetus, in different instances. He speaks particularly of a mass presented to him by Dr. Heim, in which, after very accurate examination, he was satisfied that peculiar animal life existed. The consideration of this subject, beyond an account of the opinions of the author, is foreign to our object on this occasion, which is to produce a practical article respecting the treatment of worms; and our limits will not permit us now to enter on a full discussion of it, even were we so disposed.

In the twelfth chapter, the author treats of what he terms "*pseudo-helminthen,*" by which appellation he designates those foreign bodies, animal or otherwise, which have passed from the human body during life, or have been found in it after death, and which have erroneously been supposed to be real *entozooa*, or visceral worms. Those noticed by different modern writers, to which the author confines his remarks, are the following:

1°. The *dytrachycerus, (cysticercus bicornus* of ZEDER ; the *diceras rude* of RUDOLPHI ; the *ditrachicerosoma* of BRERA.—This supposed worm was described by SULZER. Dr. Bremser, however, doubts whether the little bodies in question, observed in the feces of the patient, were worms: he is disposed to think that they were vegetable seeds which had undergone some degree of the germinating process.

2°. The *ascaris stephanostoma*, and

3°. The *ascaris conosoma.*—LENZ thus terms two bodies which BRITSCHNEIDER supposed had been evacuated from the bowels of a young man. They were probably, the author thinks, larvæ of flies.

4°. The *cercosoma*, found by CANALI in some feces, and by him regarded as a new species of intestinal worms. It was the larva of an insect; probably of the *eristalis pendulis.*

5°. The *hexathyridium venarum.*—This worm, supposed by TREUTLER to have come from a vein in the leg of a young man in the act of bathing, who was at the same time affected with ascarides vermiculares, was, in the opinion of Zeder and Rudolphi, a worm of the species *planaria,* which lives in water and sucks blood.

6°. *Dyacanthos polycephalos,* observed and described by STIEBEL* as an intestinal worm, appears, from Rudolphi's examination of it, to have been a dried portion of some vegetable-stalk, probably a raisin-stalk.

6°. *Worms in the teeth,* as certain substances which have come from carious teeth, after the use of fumigations, have been called. These, according to the author, are only the seeds of henbane or some other analogous plant, used for the fumigation, which (deprived of their capsule by having been slightly burned previously to their use) have, on escaping from the tooth, fallen accidentally into water, when, by the unequal expansion of the fibres of the germ, the seed has taken a circulatory movement, and been imposed on inaccurate observers and the vulgar as a living animal.

---

* In MECKEL's *Deutsches Archiv.* 3 B. 2 H. s. 174.

